# Assessing how global health partnerships function: an equity-informed critical interpretive synthesis

**DOI:** 10.1186/s12992-021-00726-z

**Published:** 2021-07-02

**Authors:** Katrina M. Plamondon, Ben Brisbois, Leslie Dubent, Charles P. Larson

**Affiliations:** 1 Michael Smith Foundation for Health Research Scholar, School of Nursing, Faculty of Health & Social Development, University of British Columbia, 1147 Research Road, Kelowna, BC V1V 1V7 Canada; 2 School of Health Sciences, University of Northern British Columbia, 3333 University Way, Prince George, BC V2N 4Z9 Canada; 3 Canadian Coalition for Global Health Research, 46 Cremona Crescent, Nepean, ON K2G 1A1 Canada; 4 Faculty of Medicine and Health Sciences, School of Population and Global Health, McGill University, 772 Sherbrooke Street West, Montreal, QC H3A 1G1 Canada

**Keywords:** Global health, Partnership, Equity, Evaluation, Critical interpretive synthesis

## Abstract

**Background:**

Global health partnerships (GHPs) are situated in complex political and economic relationships and involve partners with different needs and interests (e.g., government agencies, non-governmental organizations, corporations, universities, professional associations, philanthropic organizations and communities). As part of a mixed methods study designed to develop an equity-sensitive tool to support more equity-centred North-South GHPs, this critical interpretive synthesis examined reported assessments of GHPs.

**Results:**

We examined 30 peer-reviewed articles for power dynamics, equity and inequities, and contradictions or challenges encountered in North-South partnerships. Among articles reviewed, authors most often situated GHPs around a topical focus on research, capacity-building, clinical, or health services issues, with the ‘work’ of the partnership aiming to foster skills or respond to community needs.

The specific features of GHPs that were assessed varied widely, with consistently-reported elements including the early phases of partnering; governance issues; the day-to-day work of partnerships; the performance, impacts and benefits of GHPs; and issues of inclusion. Articles shared a general interest in partnering processes and often touched briefly on issues of equity; but they rarely accounted for the complexity of sociopolitical and historical contexts shaping issues of equity in GHPs. Further, assessments of GHPs were often reported without inclusion of voices from all partners or named beneficiaries.

GHPs were frequently portrayed as inherently beneficial for Southern partners, without attention to power dynamics and inequities (North-South, South-South). Though historical and political dynamics of the Global North and South were inconsistently examined as influential forces in GHPs, such dynamics were frequently portrayed as complex and characterized by asymmetries in power and resources. Generally, assessments of GHPs paid little attention to the macroeconomic forces in the power and resource dynamics of GHPs highlights the importance of considering the broader political. Our findings suggest that GHPs can serve to entrench both inequitable relationships and unfair distributions of power, resources, and wealth within and between countries (and partners) if inequitable power relationships are left unmitigated.

**Conclusions:**

We argue that specific practices could enhance GHPs’ contributions to equity, both in their processes and outcomes. Enhancing partnering practices to focus on inclusion, responsiveness to North-South and South-South inequities, and recognition of GHPs as situated in a broader (and inequitable) political economy. A relational and equity-centred approach to assessing GHPs would place social justice, humility and mutual benefits as central *practices*—that is, regular, routine things that partners involved in partnering do intentionally to make GHPs function well. Practicing equity in GHPs requires continuous efforts to explicitly acknowledge and examine the equity implications of all aspects of partnering.

**Supplementary Information:**

The online version contains supplementary material available at 10.1186/s12992-021-00726-z.

## Background

Partnerships involve two or more organizations collaborating together toward a common goal. North-South^1^ global health partnerships (GHPs) are widely promoted as a mechanism for strengthening health systems and achieving the Sustainable Development Goals [1, 2] by, for example, supporting research, providing technical assistance or service support, engaging in advocacy, or providing financing [3]. They are commonly required or incentivized by funding practices of public, private, and philanthropic institutions [2, 4]. These partnerships fall within academic and practice fields that often carry an aspirational interest in human rights and advancing equity [5, 6]. They involve different kinds of ‘partners’ (i.e., individuals or groups who make up the partnership), such as government agencies, non-governmental organizations (NGOs), corporations, universities, professional associations, and philanthropic organizations—each of which see the potential benefit and need for GHPs in their own particular ways. While many excellent examples of GHPs can be found, all contend with the realities of a long history of unbalanced North-South political and economic relationships. Vast economic and health inequities position North-South GHPs in a quagmire of inherently imbalanced distributions of resources and power.

Critical scholarship on GHPs points to the risks of exploitation and harm that can actually entrench the very inequities they seek to redress by, for example, disproportionately serving more privileged partner interests in ways that strain the already limited material and human resources of less privileged partners [7–12], or by maintaining neocolonial power structures within countries [11–13].^2^ GHPs have typically directed Northern resources, interests, and priorities toward activities carried out in the global South. Shaped by complex legacies of colonialism [14], current GHP models evolved from post-World War II international institutions that maintained “inter-colonial health systems” [15] and generally involved sending people and health-focused resources from the North to the South, against the backdrop of enormous overall Northward flows of extracted wealth [14, 15]. When neoliberal economic reforms in the final third of the twentieth century reorganized the global political economy around unregulated ‘free’ markets, reduced trade barriers, minimized welfare states, and privatization [16, 17], the landscape of global governance shifted [18]. Interest in efficiency, effectiveness, evidence, and evaluation grew and new multi-lateral funding platforms were established to funnel Northern resources to Southern recipients, usually under conditionalities [15]. Partnerships remained a central feature of universities, NGOs and international institutions, often with resources tied to official development assistance or trade.

Frequently presented as ‘good’, this flow of resources often obscures the ways in which partnerships can reinforce inequities in resources and power, ultimately serving the needs and interests of already privileged partners over the communities they serve. Equity in global health is increasingly lauded as central to advancing aspirational ideals of the field [19, 20]; yet, aligning equity-seeking intentions with action remains elusive and inconsistent [21]. Recognizing the complex sociopolitical, economic, and historical contexts in which GHPs are situated, one pathway to understanding how equity considerations are present (or not) in GHPs is through careful consideration of how such partnerships are described and assessed. In this critical interpretive synthesis (CIS), we applied an equity lens to examine what people involved in GHPs paid attention to in their partnerships, especially what they identified as important features, actions, processes, or outcomes. This review contributed to a larger mixed methods study aimed at elaborating a practical, equity-centered tool to support people involved in GHPs to navigate challenging issues of equity.

## Methods

Critical interpretive synthesis is a type of literature review that applies qualitative analysis to a systematically identified body of literature [22, 23]. Our data set was comprised of 30 articles identified in a scoping review of peer-reviewed literature published between 2010 and 2019 and indexed in any of six databases (Medline, Embase, PsycINFO, CINAHL, Web of Science, and Scopus). The scoping review, details of which are published elsewhere [24] used search terms related to global health and partnerships (including their assessment, quality, and characteristics). Articles were screened against inclusion and exclusion criteria selecting for articles that explicitly addressed a North-South partnership assessment (not a project assessment). Government-to-government partnerships were excluded. For the purposes of this review, GHPs were categorized as focusing on capacity building, research, or sustainable development (Table 1).

**Table 1:** Categorization of GHPs for the purposes of this review

Type of GHP	Description
Capacity building	Partnerships with a focus on strengthening of competencies, at the individual, community, organization or system level.
Research	Partnerships with a focus on doing (or using) research, including with graduate students.
Sustainable Development	Partnerships with a focus on advancing a development agenda and/or achieving some element of the SDGs.

Extending the descriptive findings of the scoping review, this CIS was guided by scholarship in this field that focuses on how equity considerations are reflected in the portrayal, justification, assessment, and descriptions of GHPs [12, 25, 26]. Through a series of readings, descriptive data were extracted from each article, followed by qualitative content analysis [27]. Preliminary analysis, including identifying themes, was guided by the application of six equity-centred principles for global health (19; see Table 2). Using Mendeley (an open-access reference management software program) and spreadsheets, thematic analysis was completed by two researchers (KP, BB) with experience in critical qualitative research. Elements extracted from the articles included geographic and disciplinary affiliations of authors, characterization of partnerships and partnership activities, and what authors reported as important to the functionality of their partnerships (Supplementary Table 1). Our analysis examined how equity considerations were integrated across a variety of partnerships. Rather than disaggregating our analysis by types of partnerships, themes focused on the content and practices used to assess GHPs. We considered the type of partnership in our analysis, and when this provided important contextualization of a finding, we report as such below. Interpretation of findings included attentiveness to power dynamics, issues of equity, and contradictions or challenges encountered in North-South partnerships.

**Table 2:** Application of equity-centred principles to qualitative content analysis

Equity-centred principle	Application questions integrated into analysis
Partner authentically	Do authors situate their GHP in the context of equity? How do authors discuss equity issues inherent to their GHP? How do authors assess issues of power in their partnership?
Foster inclusion	What do authors describe *doing* to address inclusion of different partners? How do authors describe responding to issues of power in their partnership? How do daily practices proactively promote the involvement and participation of people who are historically marginalized (e.g., inclusion of equity-seeking populations)?
Create shared benefits	How do authors assess the ways in which different groups are benefitting from the partnership? How are the needs of different partners understood? How was the distribution of benefits assessed?
Plan with a commitment to the future	How do authors explore long-term partnering (versus project-focused or short-term partnering)? How do authors situate the GHP in context of contributing to a more equitable future?
Respond to causes of inequities	How do authors consider and/or address the causes (and the roots of these causes) of inequities related to the issues of focus in the partnership? How do authors assess the ways in which the partnership itself can respond to causes of inequities, either in its process or outcomes?
Practice humility	How do authors explore positionality (e.g., who any one person or organization is, in the context of the work) and power in their partnership? How do authors position or describe the roles and contexts of the North and the South in the partnership? What positions of ‘knowing’ or ‘authority’ are described in the partnership? How is learning from others assessed in the partnership?

## Results

The 30 included articles spanned a wide variety of journals and disciplinary affiliations of authors (e.g., public health, global health, nursing, medicine, dentistry, health systems, anthropology, bioethics, and occupational therapy). GHPs were often described as geographically focused in a single city or region within a global South country. Some were described as multi-country partnerships funded by specific organizations, such as the Geneva University Hospitals [28] or a group of funders, such as one that involved the Bill & Melinda Gates Foundation, the Carlos Slim Foundation and the Spanish Agency for International Development Cooperation [29]. Authors reported on various kinds of North-South partnerships, involving universities, NGOs, health professional associations, and large multi-national or public-private partnership platforms. Details of data extracted for each included article are provided in Supplementary Table 1, including type of partnership, methods, authorship, clarity, and content assessed.

### How were GHPs assessed?

Roughly half of the included articles (*n* = 16) cited the use of one of twelve different evaluative frameworks. In almost all such cases, authors commented on the difficulty of knowing where to start their evaluation and often justified adaptations. Four articles presented frameworks or sets of considerations intended to guide GHPs broadly, developed through a research or GHP evaluation process [28, 30–32] and three proposed discipline-specific frameworks [33–35]. Approaches to assessing GHPs included qualitative approaches [30, 36]; literature reviews [31, 37]; mixed methods [38]; realist syntheses and evaluation [39]; grounded theory [40, 41]; social network analysis [42]; and survey research with logistic regression [43]. Descriptions of theoretical foundations varied widely, as did methodological approaches and participants (i.e., who was involved in a GHP). Interestingly, despite the shared focus on assessing *partnering*, articles directed attention to outcomes-based performance of Global South partners more frequently than the functionality of partnerships themselves.

*Authorship* of articles was of interest to us as a marker of who is granted authority or responsibility for reporting on a GHP. Authorship lists were interdisciplinary in thirteen articles [29, 34, 35, 37, 39, 42–49] and included Southern partners in sixteen articles [29, 34–37, 39, 41–44, 46–51]. Where all authors’ primary geographic affiliations could be identified (*n* = 29 articles, 152 authors), 120 (79%) authors were affiliated with institutions located in the global North - Canada [32, 35, 37, 39, 41, 44, 48, 52]; Europe (Switzerland, Ireland, the United Kingdom, Germany, the Netherlands) [30, 31, 38, 40, 45, 50, 53, 54]; the United States [29, 33, 34, 36, 49, 55–58]; Australia [51] - and 32 (21%) were located in the global South (Kenya [46], Nepal [47], Rwanda [43], and Uganda [42]).

### How did authors portray the contexts in which their GHPs were situated?

How GHPs were portrayed mattered to our analysis because it exposes assumptions underlying the relationships, structure, and character of the partnership and/or authors’ descriptions about the legitimacy and purpose of GHPs (cf, [11, 12]). Authors varied widely in how they framed the role and context of their GHPs. GHPs were often situated around a topical focus on research, capacity-building, clinical, or health services issues. Though historical and political North-South dynamics were rarely examined as influential forces in GHPs, they were frequently portrayed as complex and characterized by asymmetries in power and resources [31, 32, 34, 35, 37–40, 46–48, 50, 51], as one article stated – “relations between different actors with varying degrees of power and influence” [50]. The depth in which such asymmetries were explained also varied. In some cases, Southern deficiencies in resources simply appeared as an unexplained lack of capacity or infrastructure (e.g. India’s scarcity of occupational therapists) [52]. Others explicitly identified global health inequities as important considerations in their GHP [32, 34, 35, 37, 47, 48, 52, 55], with some arguing that responding to these inequities was central to the work of their GHP.

Three examples of equity-seeking assessments or approaches to assessing GHPs stood out in our analysis. Herrick et al. examined the complex ‘binds’ inherent to GHPs in contexts marked by deep disparities. In their ethnographic research, they found “many participants felt torn between their belief that working in partnership was the good and right thing to do and their sadness at not being able to effect the kinds of changes that were so obviously needed in such a resource-poor setting” [40]. They concluded their article by contrasting the “rosy idea of what a partnership is” [40] against the messy and inequitable realities of GHPs that tend to tolerate or overlook vast and structural inequities between partners. Murphy et al., in response to acknowledging disparities, described in-depth engagement of Southern researchers and research-users in the development of an equity- and power-focused partnership assessment framework [48]. Ridde and Capelle [32] situated North-South GHPs for research as a means of addressing issues of equity world-wide, as a response to the 10–90 gap (i.e., the inequitable distribution of 10% of research resources toward problems facing 90% of the world’s population) and the need for capacity building [32]. Their results highlighted *how* such disparities can manifest in GHPs, featuring comments from Southern researcher interviewees on the tendency of Northern researchers to ‘infantilize’ (original text “*d’une infantilisation*”) their Southern partners and emphasizing the importance of doing work that supports solutions needed by collaborators in the South [32].

GHPs were often portrayed as inherently beneficial and benevolent, with human and financial resources *necessarily* flowing from the global North toward initiatives carried out in the global South. Leffers and Mitchell [56], for example, introduced their grounded theory study by describing nurses’ “long history of service to the global community,” where nurses provide “direct nursing care to people in global settings or participate in faculty-led service-learning programs” in host settings. ‘Visiting nurses’ and ‘community host partners’ are all named without stating who they were or where they came from, leaving readers to determine (near the end of the article) that the reference to ‘community host partners’ was always in Southern settings.

In addition, the role of corporate power in GHPs was often unquestioned and legitimized, usually through acceptance or endorsement of the private sector’s role in promoting effectiveness in global health. Thiessen et al. [51], for example, justified corporate participation in a GHP by asserting that “the reason for partnering is because the private sector can achieve better efficiency through experience and innovative systems”. Problems with public-private partnerships (PPP), such as the potential for conflict-of-interest, were occasionally found [58], although without questioning the legitimacy of the PPP model. One particularly illustrative article highlights the extractive sector’s contributions to solving maternal health challenges in Papua New Guinea [51] without mentioning the same sector’s extraction of massive profits from the country and contributions to widespread environmental destruction, violence and sexual violence (c.f., [59, 60]).

### What did authors assess, or report as important to assess, in GHPs?

The specific features of GHPs that were assessed varied widely, from informally or qualitatively observed interpersonal and relational dynamics, to achievement of quantifiable population health indicators, to reporting logistics (e.g., ability to acquire funds in competitive funding environments). Authors leaned heavily toward reporting on the benefits of GHPs. Many articles reviewed literature on partnership pitfalls or failures before moving on to a success-oriented case study with little emphasis on problems, contradictions, or negative consequences. Most authors assessed aspects of partnership processes (*n* = 23), such as how decisions were made or priorities set, and a portion of these also focused on outcomes (*n* = 8). Common among all thirty articles was at least some attention to the interpersonal experience of being involved in GHPs. Many described relational concepts such as mutual trust [28, 36, 37, 42, 45, 47, 56, 57], respect, and understanding [28, 36, 47, 55] as central to their findings. Some authors directly described the value of building personal relationships and friendships in GHPs [28, 30, 39, 41, 55]. Beyond such cross-cutting relational dynamics, our analysis identified five kinds of things that authors argued were central to ‘good’ GHPs, and therefore worth paying attention to or assessing (discussed in more depth below): (a) early phases of partnering; (b) navigating issues of decision-making, process, or governance; (c) managing the implementation work of GHPs; (d) paying attention to performance and impacts; and (e) attending to issues of inclusion.

#### Early phases of partnering

Several authors found the early phases of partnering to be critical to the success of GHPs. Authors spoke to the importance of creating formal partnership agreements (e.g., memoranda of understanding) or strategic plans [28, 30, 34, 37, 48, 51, 57]. Similarly, many authors described the establishment of shared values, goals, mission, and vision early in the partnership as foundational [31, 33, 36, 39, 46, 48, 55, 57]. Many authors highlighted the need for priority setting, emphasizing the importance of alignment between the priorities of each GHP partner and local (i.e. Southern) priorities [28–30, 36, 38, 40–42, 47, 54, 56–58]. In some cases, this alignment was specifically seen as between GHPs and the national health plans of global South partners [29, 51]. In the context of GHPs for research, one article recommended feasibility studies [39] during the period of partnership building in order to assess the stakeholders’ priorities, concerns, and willingness to participate in a research partnership. Others spoke to feasibility indirectly, asserting the importance of establishing sustainable [30, 37] and sufficient resources and financing to enable the core functions of a GHP [28, 30, 38, 40, 56].

#### Navigating issues of decision-making, process, or governance

Features described as worthy of attention in GHP assessments consistently involved governance processes, which we considered to involve decision making, authority, and accountability processes within partnerships. Herrick et al. [40] described the governance and partnering processes of GHPs as inherently complex and full of contradictions. They noted that the historical shift in international cooperation for health from a focus on specific infectious diseases toward *good governance* and *partnership* was envisioned as a way to render “all sides of the relationship accountable, empowered, and responsible”, where “partnership is envisaged as both means and ends to achieving a more resilient health workforce in southern countries as well as conferring benefits on northern (or increasingly southern) partners” [40]. They examined intense conundrums, or ‘binds’, presented by the inequitable distribution of resources between the global North and South, noting, for example, that Northern funding policies and norms led via budget constraints to overlooked contextual realities, limited sustainability, and lack of responsiveness to Southern partners’ needs. Their study documented Northern partners encountering constant disappointment and frustration, as efforts in Sierra Leone were constrained by structural factors that deeply influenced partnering process and work:

…the busy and politically delicate work of trying to bind partners together around common goals—often involving endless meetings, memos, phone calls, report writing—is often far easier to articulate than the higher order and fleeting moment when everyone is bound to a shared vision and working toward promised change. When efforts to achieve means or ends fall short, as they invariably do in the Sierra Leonean context, partnership working (and especially this “busy work”) becomes experienced and enacted in ways that create a perpetual bind, a source of angst and frustration for those involved [40].

Most authors emphasized governance as central to the function and experience of partnering, with decision-making, accountability and authority tied to relational interactions. Authors consistently drew attention to the importance of clear and effective communication practices [28, 36–38, 55, 58], decision making practices [28, 38, 42, 54], role clarity [29, 39, 41, 54], and determining lines of authority, accountability, and responsibility [28, 30, 33, 36, 37, 40, 42, 47, 50, 58, 61]. Such terms were frequently named but rarely defined. One exception offered an in-depth exploration of accountability, defined as a participatory, ongoing process of holding power-holders to account [30]. In some cases, authors spoke to the importance of understanding [33, 48, 56], clarifying [32, 39, 43], or managing [40, 58] the *expectations* of organizations and individuals involved in partnering. Others emphasized the need for well-nurtured relationships, patience, and shared leadership [28, 39, 54, 55, 57]. Some highlighted the importance of evidence-informed decision-making and policies of GHPs [29, 30, 62]. Shared ownership [39, 57] and authenticity [38, 41, 48] in partnering were both mentioned in the context of discussions relevant to equity in GHPs. Sandwell et al. [44] for example, strongly emphasized the importance of relationships, reciprocity, humility, shared learning, and responsiveness to pressing healthcare services and workforce needs. Similarly, Steenhoff et al. [34] proposed that GHPs pay careful consideration to why, when, and how to partner, and who benefits from partnering. These authors, among others, found that the day-to-day behaviours, attitudes, and practices of the people involved in GHPs were instrumental in how well they worked [32, 35, 39, 41, 45, 48, 51, 52, 56, 57, 63].

#### Managing operational aspects of GHPs

Many authors assessed or emphasized the need to pay attention to the day-to-day operational work of GHPs. Authors highlighted, for example, the importance of transparency in daily communications [32, 55, 58] and in the distribution of resources [28, 33, 47, 52, 54]. Authors were attentive to management practices, such as having developed workplans, with evaluation and reporting mechanisms [30, 42, 50, 57] and a clear understanding of the day-to-day priorities [37]. Another important operational issue raised by authors was the need for fair and reasonable compensation that recognizes the contributions of staff and partners. In one study, authors emphasized the need for transparent discussion about per diems for capacity building events, identifying an uncomfortable conundrum. They argued that, while Southern partners may lose wages to participate, their professional obligations to continuing competency *should* motivate participation over expectations of per diems [40].

Some authors spoke specifically to the value of financial accountability and transparency [50]. Authors in this and comparable articles largely focused on the recipients of aid (i.e., Southern partners), implying a tendency toward unbalanced scrutiny of the outputs or performance of one set of partners. Bruen et al. [50] offered interesting insights about issues of accountability in GHPs involving relationships and actors “with varying degrees of power and influence”. They described accountability in GHPs as a participatory, ongoing process of holding power-holders to account, where power was often overlooked:

…the national and international actors who make or influence policy are largely neglected here, as are the asymmetric relations between actors that may lead to modes of accountability that are skewed to favour the interests of more powerful actors. It is necessary to also ask questions regarding who gets to decide on or design accountability interventions, to set the benchmarks or targets against which interventions or decisions should be evaluated, and whether or not efforts to improve accountability actually achieve their purported aims [50].

Generating greater accountability and equity in the context of power imbalances, they argued, requires an examination of the behaviours, influences, and actions of the power-wielders [50]. Some authors also emphasized adaptability, flexibility, and responsiveness in the implementation of GHPs [47, 57, 58], particularly in relation to on-the-ground realities of Southern settings [30, 37]. Reciprocity (e.g., in student exchanges, in resource sharing, in benefits) was an issue of significant concern for many authors [33, 36, 42, 47, 55, 57], with one large mixed-methods study reporting GHPs that have greater reciprocity tend to be of high value [46]. Several authors argued that high-value GHPs pay attention to training [56], capacity building [38, 39, 54], and mentoring [34, 38, 46].

#### Paying attention to the performance and impacts

Partnership performance and impacts were often vaguely described, without distinction between performance and benefits, or definitions of what should ‘count’ as success. No universal benchmarks or signals of ‘good’ or ‘successful’ GHPs were identified. Only one article [31] provided an explicit definition for what they believed was indicative of a good GHP, stating that “we understand ‘performance’, ‘success,’ and ‘effectiveness’ in terms of problem-solving capacity of partnerships to address the issue they have set out to solve”. Yarmoshuk et al. [46] argued that the “analysis of partnerships themselves, and their limitations, is often lacking in detail”. They questioned the legitimacy of having implementers writing about their own GHPs for the very question of competing interest and positivity biases inherent in the academic literature, “especially in an era when the use of positive adjectives such as “innovative” in academic papers has increased significantly, likely in response to the pressure to publish and need to sell results”. Though no common definition of success emerged, this literature generally inferred ‘success’ to involve benefits to individuals, organizations, and communities and offered insights about what makes GHPs work well. Most articles presented difficulties, tensions or conundrums in GHPs, with few structured mechanisms identified to support their navigation.

Overall, we found that authors paid less attention to how relationships between the Northern and Southern partners were working than on how the Southern initiative and its multiple local stakeholders were performing. For example, some authors flatly excluded the perspectives of their Southern partners, stating that “unfortunately, interviews with nurses outside the United States were limited by logistics” [56]. Without assessing the North-South partnership, authors in another article presented in-depth case study assessments of seven countries funded through the GHP, focusing exclusively on the implementation of different projects and the partnerships *within* each case [58]. More subtly, authors’ assumptions that GHPs involve a flow of resources from North to South seemed to coincide with affording greater attention to the functional activities of the partnership as carried out *in and by the Southern partners*. In another article [28] using a collaborative governance framework to guide their evaluation of a GHP, authors presented their analysis from the “Northern” perspective, describing relationships and relational concepts (e.g., ‘trust’) without including the Southern partners (who are identified as the primary beneficiaries of the partnership) in their assessment. They defend the focus on Northern voices as an effort to “further engage ‘Northern’ institutions in seeing the value of such collaborations”.

In contrast, among the authors who more directly explored relationships in GHPs, findings tended to focus on the behaviour and attitudes of Northern partners. Sandwell et al. [44] examined the *experience* of being in a GHP and emphasized the importance of long-term individual and organizational relationships, identifying ‘interpersonal rapport’ and ‘lightheartedness’ as contributors to a better partnership experience. One group of authors [38] pointed to the tendency of Northern partners to frame the benefits or successes of partnerships in terms of what they gave, whereas Southern partners were more likely to express gratitude for the opportunity (see p. 5–6). In their discussion, they return to this point, noting that Northern partners were “unable to identify many benefits to them”, presenting a challenge to sustainability of the partnership. The authors go on to suggest the need to challenge *perceptions of benefits* in the context of equity [38].

Financial sustainability and partnership expansion were echoed by some as markers of success or reasons for failure [39, 54]. El Bcheraoui et al. [29] focused on the successful achievement of quantitative indicators of maternal mortality and other health outcomes, as well as process-related “drivers of success”. Although they emphasized the value of evaluation and learning, the article is centered around fostering competition between Latin American countries, arguing that partners’ fear of losing reputational status with neighboring countries could be a novel approach to “increase the effectiveness of global health initiatives” [29]. Opportunities for career development, capacity building, and learning were frequently described as characteristic of successful GHPs. In one article, authors argued that an increase in “funds, volunteers, prestige, awards, and international visibility” [40] enabled by the West African Ebola crisis “solidified the partnership”. Visibility was also described as indicative of the value and relevance of a GHP, with authors quoting an interview participant in their large mixed-methods study [46] as saying, “because we were providing care to people with AIDS, our profile went up since we were involved in the construction of the clinics”. Herrick et al. [40] similarly criticized the concept of academic ‘success’, pointing to the visibility generated by the Ebola crisis, and its role in evoking the competitive incentive structures of academia and related institutions, in which a massive human tragedy can in fact provide career-advancing opportunities for researchers and healthcare professionals. One article [36] asserted that “using the sites to advance the U.S.-based researchers’ careers impeded success—further elaborating that “pressures or rules from funding agencies, academic institutions, and other involved parties may work against mutual benefit”.

#### Attending to issues of inclusion

The actors included as partners in GHPs varied across the 30 articles reviewed, with some authors naming organizations as the partners (e.g., international organizations, foundations and other funders and corporations, health ministries, hospitals, universities or research organizations, NGOs and other civil society organizations) (e.g., [40, 50]). Some health professions were also identified as members of GHPs, specifically medicine, nursing [33, 35, 56], midwifery [44], allied health [40], public health [49] and, in one case, health professions involved in ‘child health’ [34]. Other authors described the actors of GHPs in terms of individuals. Several articles pointed to the importance of including ‘local champions’ (implying a global South person), ‘visionary leaders’, or ‘partnership pioneers’ as key to successful partnerships [30, 42, 51, 57]. Importantly, the members of GHPs rarely included people who experienced the health issues motivating the partnership. Grassroots voices of community members or patients were largely absent from assessment of GHPs.

A relatively small number (*n* = 8) of articles explicitly addressed issues of inclusion in their GHP. Yassi et al. [39] for example, described a research partnership involving the health of healthcare workers in South Africa, in which workers’ union representatives participated in research design and planning. Njelesani et al. [41] observed that being White or Black in Zambia mattered, noting that having a name that may read as White or Black can influence relationships and participation. In their conclusion, the authors directly address power and history: “relations between the Global North (which includes countries such as Canada) and the Global South (including countries such as Zambia) are fraught with power imbalances, macroeconomic forces and colonial legacies, the partnership described here is undoubtedly influenced by, and possibly influences, such superstructure”. In another article focusing on a Canada-Dominican Republic nursing capacity-building partnership, [35] authors reported “intentionally bringing community members together to utilize their knowledge and expertise…[as] a foundational principle of community engagement”. Another article asserts that leaders of seventeen “completely indigenous” (sic) communities transmitted their priorities to the Ministry of Health [29] but neither these Indigenous leaders nor other community members were interviewed in the evaluation. Another article [57] repeatedly referred to a “vibrant consultative process” involving a range of stakeholders that did not actually include community members. In these examples, consultations by government, civil society or private sector bodies named among the formal partners often took on the role of representing grassroots voices.

## Discussion

GHPs involve active and relational process that evolves over time, are embedded in complex contexts, and evoke both organizational and personal relationships. Our analysis spanned many types of partnerships with a focus on how equity is (or could be) considered in assessing GHPs. We were not interested in whether some types of partnerships are better or worse than others, but rather in *how* the process of partnering itself could be more or less responsive to their inherent issues of equity and power. Though reviewed articles shared a general interest in partnering processes, they often lacked attentiveness to issues of equity in either process or content. Among the 14 frameworks identified to support assessment of GHPs, attention to issues of equity was limited and most placed greater emphasis on outcomes than processes. Despite most authors describing equity as something felt, or experienced, in GHPs, the *experience* of equity was not integrated as something to assess. Our findings suggest that GHPs need to balance the attention afforded to assessing processes of governance, issues of equity and inclusion, the day-to-day work and management of partnership work with bigger-picture impacts and outcomes. Although most authors discussed the importance of relationships as an interesting finding, few set out to assess relational or equity dimensions of their partnership. Integrating a relational and equity-centered approach to assessing GHPs would place social justice, humility, and mutual benefits as central *practices*—that is, regular, routine things that partners involved in partnering do intentionally to make GHPs work well. Equity-centered assessment of GHPs appears to be a shared intention, yet elusive to put into practice. Interest in the ethics of equity in GHPs extends beyond the limits of the thirty articles we reviewed, with other scholars examining inequalities and inequities in GHPs so far as to propose a code of ethics for global health collaborations [64]. The ethics of GHPs may, indeed, be framed as closely tied to how attentive and responsive they are to issues of equity.

Our analysis illuminated specific practices GHPs could embrace to enhance their contributions to equity, both in their processes and outcomes. We focus our discussion here on inclusion, attentiveness and responsiveness power asymmetries in the context of vast inequities, and recognition of GHPs as situated in a broader (and inequitable) political economy. First, with respect to inclusion, *assessments of GHPs did not include voices of all partners or beneficiaries, notably excluding those intended to benefit from GHPs and several examples where partnership assessments from only one partner’s perspective were rationalized.* The almost-complete absence of grassroots or community voices among those assessing partnerships suggests that reflection on GHPs appears to exclude the voices of the people most affected by them, and arguably those with the most legitimate role in assessing whether they are successful. The relative exclusion of community voices was met with a consistent assumption of the inherent ‘goodness’ of GHPs. Assumptions of ‘goodness’ was challenged by some [40, 46] within this literature and many in the broader field [11, 13, 65]. Greater net benefits for the Northern partners involved in GHPs were often unquestioned. Critical debates in the broader field of knowledge translation in global health increasingly invite more explicit consideration of how benefits are distributed, whose interests partnerships serve, and the importance of practices of equity, including humility, service, and shared benefits [66, 67]. Integrating equity considerations in the assessment and ongoing analysis of GHPs would draw partners’ attention to identifying imbalances in power or the distribution of benefits, and offer a platform for discussion on how these imbalances might be mitigated.

Power asymmetries, particularly in the context of extreme social, economic and health inequities were sometimes alluded to or named for the discomfort they can produce, but rarely met with practical mitigation strategies. Authors’ portrayal of GHPs as inherently beneficial for Southern partners threw into sharp focus their lack of attention to the factors that shape power dynamics and inequities (North-South, South-South). Though power dynamics are always at play in partnerships, attention to power was minimal and most articles focused more on attention to performance outcomes. Silence on the complex power dynamics at play in GHPs, and similarly, on issues of equity and inclusion is concerning.

By virtue of selecting articles reporting on North-South GHPs, all articles included in this CIS involved countries with vastly inequitable life trajectories and health outcomes shaped by the social and structural determinants of health [68] that disproportionately advantage populations that benefit from colonization over those that were colonized [14]. The need for action on the social determinants of health is increasingly acknowledged through global health organizations and governance mechanisms, such as the Pan-American Health Organization’s recent recommendations that draw attention to advancing equity in social, political and economic structures and addressing the legacies of colonization and racism [69]. GHPs operate in the broad North-South contours of colonial legacies, but are also complicated by the frequent role of Southern elite sectors in facilitating the extraction of wealth by largely-Northern interests – elite sectors which also disproportionately provide the Southern researchers and health professionals who participate in global health partnerships [11–13]. Along such lines, broader literature examining GHPs suggests that, despite frequently espousing equity ideals, they can play a role in maintaining power norms that disproportionately serve the interests of already-privileged groups, both in the Global North and in the South [11, 70, 71]. This finding evokes a commonly-noted absence of awareness of issues of power and privilege in GHPs and, by extension, a lack of capacity to engage in critical power analysis and mitigation [2, 12, 26]. Ultimately, GHPs can serve to entrench both inequitable relationships and unfair distributions of power, resources, and wealth within and between countries if inequitable power relationships are left unmitigated. Our review reinforces the need for greater capacity to do so within GHPs.

Finally, our review illuminated a disconnect between the broader political economies of GHPs and authors’ portrayals and assessments of partnering. It is crucial that those concerned with North-South inequities in GHPs have, at the very least, a general awareness of the ongoing macroeconomic drivers of such inequities, and their enormous population health impacts. Such awareness would strengthen capacity for GHPs to reduce inequities within the partnership itself (i.e., the specific people involved, in terms of opportunity and career development) and within the communities they seek to serve. Inattention to the role of macroeconomic forces in the power and resource dynamics of GHPs reveals an opportunity to extend power and equity analysis to the broader political economy in which partnerships are situated. In articles focused on PPPs, power and resource imbalances were implicitly legitimized and strengthened by the ways in which the GHPs were described. Though elevated as a mechanism for addressing resource gaps, PPPs advance a neoliberal agenda in GHPs, privileging private interests in ways that typically undermine the public sector and shift attention away from the upstream drivers of health inequities [72]. Logically, some authors described alignment between partnership goals and strategies with local priorities as important in GHPs. Yet no article examined the role of macroeconomic forces in shaping the national health plans of Global South countries, which are often subject to meeting debt conditionalities and other coercive macroeconomic mechanisms [72, 73]. Such conditionalities are, themselves, largely shaped by the priorities of international financial institutions, where the power of the Global North far exceeds that of the South. Despite GHPs’ consistent use of *alignment with national health policies* as a way of justifying the responsiveness to local communities, needs, or interests—such policies are shaped and restricted by neoliberal economic reforms that dictate limitations on government investments in health—meaning, the alignment is not *necessarily* with the global South, but rather can be an extension of Northern interests that effectively maintain an inequitable distribution of power globally [74, 75]. Future directions for study could extend the consideration of equity to mechanisms intended to evaluate technical and financial aspects of GHPs, outcomes, or accountability. Existing tools or frameworks for assessing GHPs could also could be revisited, for example with a particular interest in integrating equity considerations.

### Limitations & contextualizing results

This CIS involved a systematic search for literature reporting assessments of North-South GHPs. Our review is confined to the content authors shared in their articles, as we could analyze only what was written. In alignment with previous scholarship on promising practices for advancing equity action [12, 76], we specifically examined articles for whether and how authors situated GHPs in the context of vast economic and health inequities because we believe not doing so risks overlooking or naturalizing inequities. This decision extended an assumption that, given the distribution of power, resources, and wealth globally, North-South GHPs *should* do something to advance equity action, regardless of whether or not the partnership was established with that intention. Further, this set of articles demonstrated substantial variability in what ‘kinds’ of things were assigned value in GHPs, with few definitions offered. Publication practices and norms often restrict the attention authors can give to declaring assumptions, values, or positions, and what is reported in articles thus does not necessarily reflect the equity intentions or actions of authors [12]. Given the lack of attention to critical power analysis within a set of partnerships that involve inherent and known inequities between North and South, it is not possible to assess whether any GHPs led to inequitable or ineffective partnerships. All of these factors restricted how far our analysis could be taken while also illuminating the importance of finding ways to encourage explicit declaration of how GHPs pay attention to equity, power, and inclusion—particularly in the context of vast and long-standing global inequities in the distribution of power, resources, and wealth.

## Conclusion

GHPs are situated at a complex intersection of individual motivations, political economies, sociopolitical histories, funding and educational opportunities, and vast health and social inequities that persist within and between countries worldwide. Growing interest in issues of equity in global health extends beyond the topical focus of GHPs to include how they, themselves, function to address equity. Our findings suggest a need for tools that explicitly acknowledge and examine the equity implications of all aspects of partnering, both formal and informal. GHPs could play a positive role in shaping the determinants of equity; but only if they are done with explicit attention to issues of power and the inequities that characterize North-South partnerships. Recognizing that the root causes of health inequities, both within and between countries, involve the unfair distribution of power, resources, and wealth, issues of equity in GHPs involve making judgements about what is fair and tolerable, and what warrants mitigation. The absence of critically reflective examination of relational processes and power within GHPs points to a need for tools that can support active and equity-informed partnership by all participants, and in ways that allow and elevate voices that might otherwise be silenced.

## Supplementary Information

**Additional file 1: Supplementary Table 1.** Overview of methods, authorship, clarity, and content of included articles.

## Data Availability

The datasets generated and/or analysed during the current study are not publicly available due to the iterative nature of our analysis, but are available from the corresponding author on reasonable request.

## Abbreviations

GHP: Global health partnerships
CIS: Critical interpretive synthesis
NGO: Non-governmental organization
PPP: Public-private partnership

## Footnotes

1 In this article, we use the terms North and South to refer to global power positions: ‘global North’ or ‘Northern’ refer to countries or populations that have been principal beneficiaries of colonization, and ‘global South’ or ‘Southern’ to those that have been previously colonized. Australia, for example, is geographically situated in the Southern hemisphere, and Aboriginal populations of Australia were colonized. The country’s dominant powers and populations are beneficiaries of colonization and therefore Australia would be considered to fall into the ‘global North’.

2 Here, we use the term ‘neocolonial’ to describe persistent North-South inequities, reflecting economic and political exploitation of global South countries after the end of the formal colonial period.
