# Supplementary material for: Assessing how global health partnerships function: an equity-informed critical interpretive synthesis
Source: Global Health. 2021 Jul 2;17:73. doi: 10.1186/s12992-021-00726-z (PMC8254362; doi:10.1186/s12992-021-00726-z)
Supplement: Supplementary file 1 — Additional file 1: Supplementary Table 1. Overview of methods, authorship, clarity, and content of included articles. [file 12992_2021_726_MOESM1_ESM.docx]

**Supplementary Table 1: Overview of methods, authorship, clarity, and content of included articles**

| **First Author (Year)** | **GHP Type*** | **Description**  ***Use of GHP Assessment Framework (Yes/No)*** | **Methods**  **Framework citation (if applicable)** | **Authorship**  GN #; GS # | **Use of theory, tool or framework** | **Clear and coherent Methodology** | **Address issues of power** | **Statement of equity intention** | **Aspects of GHPs Assessed** | | | | |
| --- | --- | --- | --- | --- | --- | --- | --- | --- | --- | --- | --- | --- | --- |
|  |  |  |  |  |  |  |  |  | **Processes** | **Outcomes** | **Relationships** | **Inclusion** | **Performance of GS and/or GN partners** |
| Beran (2016) | CB  Rch  SD | Northern University partnering with Southern Health Systems, NGOs, Professional Associations, Universities  *Framework: Yes* | Leads for each of several GHP projects were presented and discussed during an annual review meeting. One author completed in-depth analysis using selected elements of a **framework for collaborative governance** (Emerson et al, 2011). Results were presented to project leads for discussion. Iterative discussions occurred during weekly meetings. Analysis and discussion provided the "Northern perspective on partnerships" (p.5 ), suggesting this is important for demonstrating the value and benefit for Northern participation in such partnerships. | GN 12; GS 0 | Yes | Yes | No | No | Yes | No | Unclear | No | GS |
| Birtch (2013) | CB  Rch | North-South research and clinical partnership between a Canadian school of nursing and a Malawian college of nursing  *Framework: Yes* | Completion of a literature review led authors to select a tool for **assessing participatory research projects**. Authors applied this measurement tool to assess 25 items of partnership, completing a qualitative assessment of meeting minutes, emails, fieldnotes, clinical journal logs, and interviews. They reported evaluation and scoring of the partnership. Methods for scoring and details about who participated in scoring is unclear. | GN 3; GS 1 | Yes | Yes | Yes | Yes | Yes | Yes | No | No | GS + GN |
| Bruen (2014) | SD | Multi-lateral public-private partnership with Northern 'funders' and Southern 'recipients'; multi-stakeholder board  *Framework: No* | Authors discuss the Global Fund for AIDS, TB & Malaria as an example for assessing GHPs, including changing dynamics of global health cooperation, changing dynamics of accountability, and accountability mechanisms. | GN 2; GS 2 | No | No | Yes | No | No | No | No | No | GS + GN |
| Buse (2011) | SD | Multi-lateral partnerships focused on product access or product development; some global coordination and financing mechanisms  *Framework: No* | Methodology unclear.  Authors synthesize eight public-domain evaluations of GHPs alongside reflections from their experiences and on several research projects. | GN 2; GS 0 | No | No | No | No | Yes | Yes | Yes | Yes | GS |
| Citrin (2017) | CB  Rch | Multi-Partner partnership global health academic partnership, includes Nepal Ministry of Health, Nepal NGO, US-based University  *Framework: Yes* | Methodology unclear. Review the quality and effectiveness of the GHP using **Tropical Health & Education Trust (THET) Principles of Partnership Framework**. | Unclear | Yes | No | Yes | Yes | Yes | No | Yes | Yes | GS |
| Coffey (2018) | CB  SD | Multi-agency healthcare delivery partnership, including industry (pharma), academia, NGOs, UN agencies, and donors--Implementation focus  *Framework: Yes* | Use the "empirically validated" measure that "can be used to measure the relative effectiveness of a successful collaborative effort to implement a new program or policy" (p. 179): **Brown's Coalition Functioning Framework**, applied to a mix-methods case study (semi-structured interviews and document analysis). The data gathering was done by an independent consultant. | GN 3; GS 0 | Yes | Yes | No | Yes | Yes | No | Yes | No | GS |
| Dean (2015) | CB  Rch | GHPs between Northern and Southern universities, supported by a funding scheme to facilitate research capacity building and exchange between UK and African universities  *Framework: No* | Mixed-method retrospective evaluation, using cross-sectional survey, semi-structured interviews and focus group discussions. Participants were purposively sampled with an intention to achieve maximum variation in geographic location, sex, seniority, and discipline. | GN 4; GS 0 | No | Yes | Yes | No | Yes | Yes | Unclear | Yes | GS |
| El Bcheraoui (2017) | SD | Salud-Mesoamérica Initiative, a results-based aid partnership, funded by BMGF, IDB, SAIDC to fund initiatives related to maternal and child health in Central America and Chiapas, Mexico.  *Framework: Yes* | Grounded theory approach using document review, key informant interviews, focus groups, and a partnership analysis. Used the **PARTNER tool** (online tool for purchase; for 'assessing networks') to demonstrate network connections and trust. | GN 8; GS 1 | Yes | Yes | No | No | Yes | Yes | No | No | GS |
| Herrick (2018) | CB  SD | Partnership between King's College London and Sierra Leone, involving a Northern University partnering with many Southern partners from one country  *Framework: No* | Methods described as “ethnographic” (p.524), using 30 interviews that drew on experiences in GHPs from international development to global health. | GN 2; GS 0 | No | No | Yes | No | Yes | Yes | No | No | GS |
| John (2016) | Rch | General research partnerships between Northern Universities & Southern partners  *Framework: No* | Open ended questions to five global health research colleagues from Kenya, Uganda, and US. | GN 1; GS 2 | No | No | No | No | No | No | Yes | No | GS + GN |
| Kamya (2017) | SD | Public-private partnership, GAVI, as an example of global health governance; focus on GAVI partnership in Uganda  *Framework: Yes* | Used **GAVI Partners' Engagement Framework** as a mechanism to illustrate linkages between inputs and outcomes, mixed-methods case study design, with document review and in-depth interviews and analysis involving social network analysis and a case study (Uganda); and also use **Brinkerhoff's framework** (5 points to assess partnerships). | GN 3; GS 3 | Yes | Yes | No | No | Yes | No | Yes | Yes | GS |
| Leffers (2010) | CB  SD | Northern academic and professional nurses going to Southern countries for research, education, electives, practice, or missions  *Framework: No; authors develop a theory* | Grounded theory study, drawing from 13 interviews with "nurse experts' from the US, described as individuals with many years of experience working in 'global health' | GN 2; GS 0 | Develop one | Yes | No | No | Yes | No | Yes | No | GS + GN |
| Lipsky (2016) | SD | Focuses on Uganda's costed implementation plans for family planning, involving Uganda ministry of health, NGOs, UNFPA, and USAID  *Framework: Yes* | "The purpose of this evaluation was to look at the rules of engagement and relationships within the partnership that developed Uganda’s CIP." (p. 286), applying **Brinkerhoff’s framework** for assessing and improving partnerships, which assesses Pre-requisites for success, degree of partnership, outcomes, partner performance, efficiency and strategy. Using a "deductive qualitative approach" and semi-structured interviews. | GN 4; GS 0 | Yes | No | No | No | Yes | No | Yes | No | GS |
| Murphy (2015) | CB-Rch | Northern academic partners and Southern researchers or research users from multiple settings  *Framework: No; authors develop a framework* | Sequential consultation process focused on learning, with each consultation a 3-day multi-stakeholder workshop style event (Africa, South Asia, Latin America). Methods involved pre-workshop engagement and post-workshop follow-up. | GN 3; GS 1 | Develop one | Yes | Yes | Yes | Yes | Unclear | Yes | Yes | GS + GN |
| Ndgenga (2016) | CB | US academic partners working with Rwandan researchers or research users (some academic, some from Ministry of Health)  *Framework: No* | Online administration of 85-item questionnaire for US faculty and 71-item questionnaire for Rwandan faculty were administered (different instruments for US and Rwandan faculty), followed by regression analysis. | GN 4; GS 4 | No | No | No | No | No | Yes | No | No | GS + GN |
| Neuhann (2017) | Rch  SD | German Development Agency, German universities, Malawian hospitals, focused on knowledge transfer and reciprocal exchange visits  *Framework: Yes* | Document analysis using **Capacity WORKS model** and survey that was built on the Partnership Evaluation Guidebook promoted by the German Centres for Disease Control & Prevention | GN 2; GS 0 | Yes | No | No | No | Yes | Yes | Unclear | No | GS + GN |
| Njelesani (2013) | Rch | Northern university and researchers with Southern Researchers & NGO, with applied health sciences focus  *Framework: Yes* | Apply the **Global Health Research Initiative Framework** (Boutilier et al, 2011) retrospectively to the partnership using "collaborative and candid dialogue" about four considerations: (1) Long-term and sustainable north–south partnerships (2) Interdisciplinary responses to complex issues (3) PAR that grounds the research in its context (4) Research with a policy or practice impact orientation | GN 4; GS 1 | Yes | Yes | Somewhat | No | Yes | No | Yes | No | GS + GN |
| Pattberg (2016) | SD | Multi-stakeholder partnerships for sustainable development, generally  *Framework: No; authors develop nine considerations* | Methodology unclear.  Findings were "distilled from the literature" and involved consultation with many major international civil society organizations. The article's first two figures refer to two other studies where the partnership evaluation appears to be conducted (not clearly stated). | GN 2; GS 0 | Develop one | No | Yes | No | Yes | Yes | No | Yes | GS + GN |
| Pérez-Escamilla (2014) | Rch  SD | School-based public-private partnerships in multiple countries, focused on reducing obesity  *Framework: Yes* | "Qualitative analysis" of documents and key informant interviews, using **National Institutes of Health 12 guiding principles** to maximize the effectiveness of PPPs in nutrition research (Alexander et al., 2015), which focus on pre-requisite principles, governance principles, and operational principles. | GN 1; GS 0 | Yes | No | No | No | Yes | Yes | Yes | Yes | GS + GN |
| Ramaswamy (2016) | SD | Northern university - Southern-focused NGO that supports clinical partnerships, training, and development in a number of different countries.  *Framework: Yes* | Case study methods, with research question: "gain a deeper understanding of how the partnership model works in various contexts, and what aspects of the model should be emphasized in different settings, using document analysis, interviews, focus groups, and clinical outcomes data. Did a review of literature for partnership phases with the **"Kybale" model of partnership** (Kybale is the non-governmental organization involved). | GN 3; GS 2 | Yes | No | No | No | No | Yes | No | No | GS |
| Ridde (2011) | Rch | A multi-institutional initiative, Canadian-based with 4 universities in Quebec, to study the challenges of North-South partnerships for global health  *Framework: No* | Exploratory qualitative study using focus groups with 6 researchers each from both North and South, selected for their experience in N-S partnerships for health. | GN 2; GS 0 | No | No | Yes | Yes | Yes | No | Yes | No | GS + GN |
| Sandwell (2018) | CB  SD | Twinning partnership between a Canadian and Tanzanian professional association and universities  Framework: No | Methodology unclear.  Authors provide a discussion about why professional associations matter; the benefits of twinning in GHPs; and offer an in-depth discussion of the partnership and what made it succeed. | GN 6; GS 3 | No | No | No | No | Yes | Unclear | Yes | No | GS + GN |
| Sriharan (2016) | CB  SD | Northern academic institutions with Southern hospitals, focused on continuing medical education of health professionals in GS  *Framework: No; authors develop a theory* | Two-part realist approach involving a realist review and interviews/ | GN 4; GS 0 | Develop one | Yes | No | No | Unclear | No | Yes | No | GS + GN |
| Steenhoff (2017) | CB  SD | A network of Northern pediatricians from engaged in a collaborative initiative with Southern partners for mutual benefit in global child health partnerships  *Framework: No* | Methodology unclear.  Literature review completed collaboratively by an expert panel. No specific literature review methodology or search criteria are provided. | GN 10; GS 3 | No | No | Yes | Yes | Yes | Yes | Yes | Yes | GS + GN |
| Storr (2018) | CB  Rch | Canadian university programs in occupational therapy partnering with Southern countries for "ethical and sustainable fieldwork experiences" (p. 34) (by Canadian students in the South, where OT not well established)  *Framework: Yes* | Methodology unclear.  Completed a program evaluation using **Working Group for Ethical Guidelines on Global Health Training (WEIGHT)** guidelines to guide critical reflection. | GN 12; GS 1 | Yes | No | No | Yes | No | Yes | No | No | GS + GN |
| Thiessen (2018) | CB  SD | Public–private partnership between the National Department of Health in Papua New Guinea, Government of Australia, and the Oil Search Foundation to train workers in essential and emergency obstetric care  *Framework: No* | Methodology unclear.  Report using qualitative methodology, including interviews and focus groups (n=85 selected from a set of 219), gathered over a 3-year period. | GN 3; GS 1 | No | No | Yes | No | Yes | No | Yes | No | GS |
| Underwood (2016) | CB | Partnership between Canadian university and Southern partners in the Dominican Republic and Haiti, offering 'placements' for midwifery and nursing students for an international elective for community-based learning  *Framework: Yes* | Descriptive, exploratory, qualitative method, using **Leffers and Mitchell framework** (Leffers & Mitchell, 2011) to gather narrative data about the perspective of partners from 23 partners in the Dominican Republic and Haiti (described as ‘host partners’). | GN 4; GS 1 | Yes | Yes | Yes | Yes | Yes | Unclear | Yes | No | GN |
| Upvall (2018) | CB  SD | Research study looking at Southern perspectives on North-South partnerships that involve Northern nursing students doing international electives in Southern settings  *Framework: Yes* | Grounded theory, using semi-structured interviews with 15 Southern participants from a diversity of countries, 14 with more than 5 years' experience in GHPs, using semi-structured interviews. Authors report efforts to uphold quality standards in grounded theory. | GN 2; GS 0 | Yes | Yes | Unclear | No | Yes | No | Yes | Yes | GN |
| Yarmoshuk (2018) | CB | International inter-university partnerships between Northern universities and Sub-Saharan African universities, with a focus on capacity building of health professionals and health professional programs in African universities  *Framework: Yes* | Mixed methods, using quantitative analysis of 125 distinct partnerships previously mapped into higher-, middle-, and lower value partnerships to determine the characteristics that contributed to the partnerships' value. Key informant interviews and focus groups were conducted in 4 universities, each with 15 -28 respondents (total n=88 Global South), in medicine, nursing and public health. Another round of interviews followed (n-59, global north). No specific qualitative pr theoretical approach is named. **Kernaghan's classification of partnerships** is applied in the analysis | GN2; GS 3 | Yes | Yes | Yes | No | Yes | No | Yes | No | GS + GN |
| Yassi (2014) | CB-Rch | Canadian research partnerships with a South African research program to improve the health of health workers in Free State, South Africa, especially with respect to morbidity and mortality associated with tuberculosis and HIV  *Framework: Yes* | Narrative qualitative approach, using feminist, Indigenous, and post-colonial perspectives, using iterative framework for complex randomized controlled trials | GN 6; GS 3 | Yes | Yes | Yes | No | Yes | Unclear | Yes | No | GS + GN |

*CB=capacity building; SD=sustainable development; Rch=Research

**GN=Global North; GS=Global South

**Table 1a: Acronyms appearing in Supplementary Table 1**

| BMGF | Bill & Melinda Gates Foundation |
| --- | --- |
| IDB | Inter-American Development Bank |
| GHPs | Global health partnerships |
| GN | Global North |
| GS | Global South |
| NGO | Non-governmental organization |
| SAIDC | Spanish Agency for International Development Cooperation |
| UNFPA | United Nations Population Fund |
| UK | United Kingdom |
| US | United States |
| USAID | United States Agency for International Development |
